# Notes and News

**Published:** 1888-12-15

**Authors:** 


### Notes and News.

Collected and Communicated.

The Lord Mayor presided at the annual meeting of the Surgical Society on December 7th, and expressed his admiration of the charitable societies of the metropolis. The subscriptions last year amounted to nearly four thousand pounds, and eleven thousand appliances were given to the afflicted.

Lady Dufferin's last public acts in India were to lay the foundation-stone of the Dufferin Zenana Hospital, and to receive in solemn state 700 native ladies, who desired to present an address. For the proper carrying out of the last, Government House had to be converted for awhile into a jealously-guarded Zenana, and even the Viceroy himself had to leave the precincts during the impressive and grateful eremony.

On December 6th the Countess of Chesterfield laid the foundation-stone of a new eye and ear hospital for Hereford. The building, which will be of very beautiful design, will cost £2,500, and of that amount £1,800 already has been subscribed. The patients will not be confined to the county of Hereford, but will come from Monmouthshire and Breconshire as well. A short service was conducted by the Dean of Hereford, and then a collection, amounting to several hundred pounds, was made.

A clever article on "How to be Ill," which appeared in the *Globe* lately, might be read with great profit by many ladies, with whom this is the sole subject of study. To many of us illness is a thing to dread, and disease is regarded with horror; but still there are some women who are never happy unless they have an excuse for a daily call from the doctor, and who love to recline on a sofa wrapped in a becoming shawl. To be an invalid on whom sympathy is showered, and on whom a whole family and numerous friends attend, may be supposed to have some drawbacks in the restraints and penalties which such a position imposes. A man is apt to become querulous and morose under such circumstances, and there is this at least to be said for invalid ladies, that they know "How to be Ill" much more gracefully and agreeably than the stronger sex.

Two cases of lockjaw were reported in last Friday's papers, the first that of a boy named Eli Foster, whose parents live at 1, King's Place, Brentford. While at play a brick fell upon the boy's finger. The injury, though thought slight of at the time, brought on tetanus, and a painful end. The second case was more singular, and the victim died at King's College Hospital. At the inquest it was stated that on November 24th the deceased was engaged in unloading a van of manure, when a piece of steel, which had by some means got mixed with it, penetrated the second finger of his left hand. He was attended by a medical man, but symptoms of lockjaw set in, and he was removed to the hospital, where the finger was amputated. Deceased got worse, and died on December 4th. Mr. Edgar White, house surgeon, stated that death was due to lockjaw, consequent upon his injury. A verdict of Accidental death was returned.

Surgeon-Major John Ince, M.D., of The Mount House, Farningham, Kent, is a candidate for a seat on the Kent County Council for the representation of the Dartford (No. 2) Division. There are already two other candidates in the field. It is beyond our province to try to influence political or other elections; but we may be permitted to say that Surgeon-Major Ince is a man of undoubted honesty and courage, and of some experience in public affairs.

Sir Morell Mackenzie delivered a lecture at the Edinburgh Philosophical Institute last week, on "Speech and Song." In commencing Sir Morell Mackenzie observed: "He accepted their invitation with some hesitation, for when he received it he was at Charlottenburg, in a position of unexampled difficulty and responsibility. He could not say that he was particularly sensitive to criticism, but it was gratifying to him to find that, whatever others might say, in 'dear old Scotland,' the home and cradle of his forbears, his countrymen stood by him."

Contrary to expectation, Mr. Alfred L. Cohen's proposal referred to last week, was, we are glad to say, unanimously adopted by a large meeting of the representatives of the clergy and congregations contributing to the Hospital Sunday Fund, which was held at the Mansion House on Monday, the Lord Mayor presiding. Mr. Nelson Hardy, F.R.C.S., again contended that the awards made to the South London hospitals and dispensaries, when compared with the sums received from the South London congregations, were altogether inadequate and unfair, a statement which was warmly repudiated by a representative of the South London clergy and ministers, who spoke subsequently. Mr. Hardy intimated that his onslaught was made after consultation with the Superintendent of Guy's Hospital; but his intervention only caused an attack to be made upon the administration of Guy's, and resulted in the production of evidence that that hospital had been treated with exceptional favour, looking to all the circumstances of its position. It is satisfactory to note that Sir Sydney Waterlow was able to state: "The Council had distributed a larger amount of money this year than at any previous time, and that the collections were the largest that had yet been made, though in 1887 the Fund was increased by a legacy of £1,000."

The discussion on charity appeals which has been going on in the *St. James's Gazette*, has at last drawn to a happy conclusion. The secretary of the Charity Organisation Society declares he has done his best to aid the benevolent by giving lists of worthy and unworthy charities, but he states that it is difficult to get detailed information, which is often refused by shady institutions. Of course his opponent, who signs himself J. H. L., declares that unless information of a precise and compatible kind is given of the work and funds of any charity it ought not to be supported, an opinion with which we must all agree. Then a third correspondent comes on the scene, who signs himself J. G. M., and tells where just such information will be shortly attainable, namely, in the "Hospital Annual of 1889." Another amusing fact in connexion with the correspondence is brought out by Mr. Brabrook, of the Registry of Friendly Societies, who states in Tuesday's *Times* that all societies having any benevolent or charitable purpose may be registered under the Friendly Societies Acts, as Mr. Lock desires; but when this was urged upon Mr. Lock some time ago with respect to the Charity Organisation Society itself, the committee of that society resolved not to become registered. This is entertaining and instructive reading, for it seems to show that the council of the C. O. S. at any rate do not consider "what's sauce for the goose is sauce for the gander." But Mr. Lock, judged by his letters to the papers, must surely be himself in favour of registration, whatever his council may decide. Is this not the case?

THE following vacancies are announced this week, the amount of salary in each case being given after the name of the institution :—

**Resident Medical Officers.**

West London Hospital, Hammersmith. House Physician and House Surgeon.—Board, etc.
Queen's Colleges, Ireland. Professor of Medicine.
Argentine Republic. Surgeon.
Stockport Infirmary. Assistant Medical Officer —£70, board, etc.
Miller Hospital, Greenwich. Senior Medical Officer.—£60, board, etc. Also Junior Medical Officer.—£30, board, etc.
Portsmouth Lunatic Asylum. £120, board, etc.
Brompton Consumption Hospital. Assistant Physician.
Stamford Hill Dispensary.—£105, fuel, etc.

**Non-resident Medical Officer.**

Township of Toxteth Park. Assistant Medical Officer.—£100, house, etc.

THE Coventry and Warwickshire Hospital, in issuing its annual report, proves its efficiency by demanding more accommodation. Patients have to be refused admmission, and the nurses and servants' quarters are uncomfortably crowded. Last year 511 in-patients and 3,573 out-patients were treated, at a total cost of £2,968. The cost per bed is £24.

MISS AGNES WESTON has issued a most artistic plea for her work in the Navy, in the shape of a beautifully illustrated little book, called "Shaking-out a Reef." It is thirteen years now since the Sailor's Rest was opened at Devonport and the very first year 15,000 of our Blue-jackets slept under its roof; but last year at Devonport and Portsmouth 80,000 were accommodated on the premises, whilst many more had to be turned away because there was absolutely no room to take them in. The many people who are so fond of vociferating that they "all love Jack" can now give practical form to their declaration by helping Miss Weston to "shake-out a reef," or enlarge her premises. A "cabin" only costs £30, and the Empress Frederick has given one as an example which others may follow.

THE chairman and secretary of the Metropolitan Hospital have written to the *Times* to explain the provident principle on which that institution now works. Those amongst us who can remember the year 1873, and the dismissal of Dr. John Chapman from his post of assistant physician to the old Metropolitan Free Hospital, simply because he advocated reforms now recognised as necessary by all, may well ponder on the mutability of human affairs. Doubtless Dr Chapman, in his retired home in Paris, looks across at his own country which has so illtreated him, and is well pleased to see his doctrines grown so popular. But the past is never quite dead to us, and the memory of old wrongs is bitter till the hand of friendliness is extended again. Does the Metropolitan Hospital of to-day acknowledge with gratitude its indebtedness to Dr. Chapman?

A WRITER in the *Leeds Mercury* gives an account of the Leeds Public Dispensary, and at the same time appears the report of the Tunbridge Wells Provident Dispensary. It is useful to contrast the two, and see how admirable is the provident system compared with the free system. At Leeds, in spite of all endeavours to the contrary, the charity is greatly abused, and wealthy people send their servants there for advice. The committee regrets to announce a balance against the treasurer of upwards of £300. Now the balance sheet of the Provident Dispensary at Tunbridge Wells reads: Receipts, including £127 11s. 6d. subscriptions and £5 10s. 6d. donations, and £541 9s. 9d. members' payments and fines, total £679 6s. 2d.; while the chief items of expenditure were £74 5s. 7d. to drugs and carriage, £40 rent, £90 salary, and £406 18s. 11d. divided among the medical officers, leaving a good balance in favour of the treasurer. And the report shows also an increase of both subscribers and members.

THE last volume of the ninth edition of the *Encyclopædia Britannica*, which has lately been published, contains two articles of great interest to all concerned in medical subjects: The first, an article on "Vaccination," by Charles Creighton, M.D.; and the second an article on "Yellow Fever," from the same pen. In the article on "Vaccination" Dr. Creighton, after an historical sketch, boldly tells the truth anent the risks inherent in cow-pox infection, which he discusses under five heads—erysipelas, jaundice, skin-eruption, vaccinal ulcers, and so-called vaccinal syphilis. Facts and figures as to these after evils are plentifully provided. Turning to the influence of vaccination upon small-pox, Dr. Creighton decries its utility either to the individual or to the State, and gives numerous tables to support his views. Revaccination is however summarily dismissed as an unimportant precaution.

IN the article on "Yellow Fever," the chief point of interest is the theory of the disease advanced by Dr. Creighton, who founds his deduction on two facts, (1) that yellow fever in time and place has dogged the steps of the African slave trade, and (2) that the African negro has a large racial immunity from the fever. Dr. Creighton argues that, owing to physiological differences, the discharges from the negro body may become, by their effluvia, specifically poisonous to white men under special circumstances. Slaves suffering from nostalgia, despair, and the sense of wrong, pass on to those who wrought their sufferings an epidemic which chiefly affects the liver, and stands, in fact, for a total arrest of the hepatic functions, biliary and otherwise. Surely this theory of nature's retribution is more startling than any theological doctrine of punishment for sin.

NOT far out of Manchester, at Mauldeth, there is a large Hospital for Incurables containing 100 patients, but strangely enough, none of them suffering from cancer. Some time ago plans were prepared for cancer wards, but were laid aside in favour of other matters. This seems a mistake, for there is no incurable disease attended with so much suffering to the victim and unpleasantness to the friends as cancer, and in its malignant forms isolation should always be insisted on. Also sufferers from cancer shrink from making known their complaint till the latest moment, so that the usual long waiting for election is not appropriate to their cases. Special wards, to which immediate admittance can be obtained, should be reserved in every Home for Incurables for these sad cases. Surely some of the rich Manchester manufacturers must have had acquaintance with this disease, and, in compassion for their poorer brethren afflicted with such horrors, will provide for them a place of retreat.

A NEW Girls' Home, at Woodford, Essex, was opened by the Rescue Society on November 29th with a dedication service, presided over by Mr. J. Gard, the chairman and treasurer to the society, and other gentlemen addressing the meeting. Mr. C. Stuart Thorpe, the secretary, read some interesting figures of the cost of the new buildings, furnishing, etc., by which it appeared that from the insurance and subscriptions the whole of the cost had been received except about £200, for which an earnest appeal was made to enable the new home to start free from debt. The Knighton Girls' Home, which the new building is called, has been erected on the site of the old home burnt down in January last, and is designed to accommodate 45 girls, the cost being about £1,800 exclusive of furnishing. The designs were prepared by Mr. Edward Tidman, C.E., M.S.A., of Westminster, who was presented by the Chairman, on behalf of the committee, with a silver salver during the evening, in acknowledgment of his honorary services in designing and superintending the erection of the new home.

